# Examining immune-inflammatory mechanisms of probiotic supplementation in depression: secondary findings from a randomized clinical trial

**DOI:** 10.1038/s41398-024-03030-7

**Published:** 2024-07-24

**Authors:** Lukas Sempach, Jessica P. K. Doll, Verena Limbach, Flavia Marzetta, Anna-Chiara Schaub, Else Schneider, Cedric Kettelhack, Laura Mählmann, Nina Schweinfurth-Keck, Mark Ibberson, Undine E. Lang, André Schmidt

**Affiliations:** 1 Translational Neuroscience, Department of Clinical Research (DKF), University of Basel, Basel, Switzerland; 2 University Psychiatric Clinics Basel (UPK), University of Basel, Basel, Switzerland; 3 Vital-IT Group, SIB Swiss Institute of Bioinformatics, Lausanne, Switzerland; 4 Translational Psychiatry, Department of Clinical Research (DKF), University of Basel, Basel, Switzerland; 5 Experimental Cognitive and Clinical Affective Neuroscience (ECAN) Laboratory, Department of Clinical Research (DKF), University of Basel, Basel, Switzerland

**Keywords:** Depression

## Abstract

We recently indicated that four-week probiotic supplementation significantly reduced depression along with microbial and neural changes in people with depression. Here we further elucidated the biological modes of action underlying the beneficial clinical effects of probiotics by focusing on immune-inflammatory processes. The analysis included a total of N = 43 participants with depression, from which N = 19 received the probiotic supplement and N = 24 received a placebo over four weeks, in addition to treatment as usual. Blood and saliva were collected at baseline, at post-intervention (week 4) and follow-up (week 8) to assess immune-inflammatory markers (IL-1β, IL-6, CRP, MIF), gut-related hormones (ghrelin, leptin), and a stress marker (cortisol). Furthermore, transcriptomic analyses were conducted to identify differentially expressed genes. Finally, we analyzed the associations between probiotic-induced clinical and immune-inflammatory changes. We observed a significant group x time interaction for the gut hormone ghrelin, indicative of an increase in the probiotics group. Additionally, the increase in ghrelin was correlated with the decrease in depressive symptoms in the probiotics group. Transcriptomic analyses identified 51 up- and 57 down-regulated genes, which were involved in functional pathways related to enhanced immune activity. We identified a probiotic-dependent upregulation of the genes ELANE, DEFA4 and OLFM4 associated to immune activation and ghrelin concentration. These results underscore the potential of probiotic supplementation to produce biological meaningful changes in immune activation in patients with depression. Further large-scale mechanistic trials are warranted to validate and extend our understanding of immune-inflammatory measures as potential biomarkers for stratification and treatment response in depression. Trial Registration: www.clinicaltrials.gov, identifier: NCT02957591.

## Introduction

With a global lifetime prevalence of approximately 25%, major depressive disorder (MDD), poses significant individual and societal burden [1, 2]. Antidepressants bring symptom relief, but over 50% of cases do not respond to initial treatment, with one in three patients showing resistance to antidepressants [3]. The limitations of current treatments, in light of the global impact of MDD, underscore the urgent need for alternative treatment approaches. Probiotic interventions targeting the gut microbiota show promise in relieving depressive symptoms [4]. Multiple randomized controlled trials (RCTs) demonstrated a beneficial effect of probiotic supplementation on depressive symptoms in MDD patients [5–8], including our own trial [9]. However, a significant research gap remains in understanding the specific biological mechanisms underlying the beneficial clinical effect of probiotic supplementation in MDD. Such mechanistic analyses aim to identify biological targets for patients’ stratification and developing more efficient and tailored microbial interventions.

Probiotic supplements are formulations of living microorganisms that provide a health benefit through modulation of the microbiota. They are thought to positively impact mental health, via altering the microbiota gut brain (MGB) axis [10], a set of bi-directional communication pathways between the gut and the brain, including endocrine, immune and neurotransmitters systems [11]. Dysfunctions of the MGB axis, alongside compositional and functional (e.g., metabolomic and transcriptional) changes in gut microbiota, known as dysbiosis, have been detected as important factors in the pathology and treatment of depression [12]. In patients with MDD, gut dysbiosis is associated with a disrupted gut microenvironment, harming the protective functioning of the gut epithelium which leads to intestinal barrier dysfunction [13]. The damaged intestinal barrier (commonly referred to as “leaky gut”) allows increased systemic translocation of gut metabolites, microbial cell components, or even the microbiota causing a range of negative consequences that have been implicated in the pathogenesis of depression [14–16].

Psychiatric disorders, including depression, exhibit a transdiagnostic pattern of gut microbial disarray, marked by a distinct pattern of depleted anti-inflammatory and enriched pro-inflammatory bacteria [17]. This pro-inflammatory microbial state is particularly noteworthy for depression, as chronic low-grade inflammation is a known pathologic feature of depression [18]. Approximately 1/3 of patients with depression have elevated immune-inflammatory markers [19], and a pro-inflammatory state is a common feature of no-responders to antidepressant medication [20, 21]. In animal models, probiotics were found to counter gut microbiota perturbation by increasing beneficial bacteria and improving overall microbial diversity, causing a reduction in circulating immune-inflammatory markers [22, 23]. But whether probiotics can produce similar reparative effects on dysbiosis and inflammatory mechanisms in patients with depression, and whether these changes can have antidepressant effects remains an area of limited exploration. A comprehensive meta-analysis on the impact of probiotic supplementation on immune-inflammatory markers in a clinical population did report reductions in a range of cytokines, including high sensitivity C-reactive protein (hs-CRP), and interleukin 6 (IL-6), while showing no effects for other markers including interleukin 1β (IL-1β) [24]. However, these implications are limited due to the diverse patient groups and their physiological states. In terms of immune-inflammatory mechanism of probiotics in depression only few RCTs assessed cytokines. Some studies found that probiotic supplementation reduces hs-CRP [8], and decreases IL-6 gene expression levels [25], although other studies reported no changes for these cytokines [26–28].

Hyperactivation of the hypothalamus–pituitary–adrenal (HPA) axis is a key factor linked to the pathophysiology of depression and inflammation-related gut microbiota alterations [12]. Animal models of chronic stress triggering the HPA axis, demonstrated negative effects on the gut microbiota composition and increased IL-6 levels [29]. Studies in healthy participants also indicate that psychosocial stress reduces gut microbiota diversity and beneficial microbes [30]. Probiotics may counter these effects and exert anti-inflammatory changes by modulating the gut hormone ghrelin and the peptide hormone leptin. Alterations in the gut microbiota affects ghrelin secretion and signaling [31], with specific gut bacteria affecting leptin and ghrelin levels [32, 33]. Ghrelin and leptin in turn interact with the HPA axis and influence immune-inflammatory markers. In healthy participants, exposure to a stress test increased stress ratings and serum cortisol, associated with ghrelin secretion [34], while injections of ghrelin enhanced plasma cortisol [35]. Leptin, conversely, inhibits the HPA axis. Injections of leptin in rats decreased adrenocorticotropic hormone (ACTH) levels [36], and HPA axis activity in humans reduced leptin but increased ACTH and cortisol levels [37]. Moreover, both ghrelin and leptin were found to modulate immune-inflammatory pathways linked to depression. Ghrelin injections reduced IL-6 levels in rats, an effect diminished by vagotomy, indicating that ghrelin down-regulates pro-inflammatory cytokines through vagus nerve activation [38]. Similarly, in humans, ghrelin was found to inhibit IL-6 and IL-1β [39]. In contrast, gastric leptin levels correlated positively with proinflammatory cytokines such as IL-6 and IL-1β, in patients with Helicobacter pylori infection [40]. Furthermore, leptin directly induced the secretion of IL-6 and IL-1β by human T cells [39]. In sum, changes in the gut microbiome may directly influence systemic immune-inflammatory processes, with ghrelin and leptin playing crucial roles in modulating both endocrine (HPA axis) and immunological (inflammation markers) pathways of the MGB axis.

We recently performed an RCT investigating probiotic supplementation in individuals with depression [9]. Primarily, we found that the intervention alleviated depressive symptoms (d = 0.62) and positively affected the gut microbiota composition. Here, we present additional analyses that further uncover the biological mechanisms underlying the positive impact of probiotic supplementation on depressive symptoms. Specifically, we report serum concentrations of (1) immune-inflammatory cytokines (IL-1β, IL-6, CRP, MIF), and (2) gut-related hormones (ghrelin, leptin), (3) saliva concentrations of cortisol, (4) transcriptional (gene expression) changes, and (5) subjective appetite measures.

## Patients and Methods

This is a secondary analysis of a double-blinded RCT of probiotic supplementation in patients with depression (NCT02957591, www.clinicaltrials.gov). Clinical, microbial, and neural findings have previously been published [9, 41, 42], indicating beneficial effects of the probiotic intervention on depressive and cognitive symptoms, gut microbiota composition and fronto-limbic brain structure and function. Here we further explored probiotic effects on immune-inflammatory mechanisms.

### Participants

Adult inpatients (n = 60; 18–65 years of age) with a current depressive episode (F31.3-F34 according to ICD-10 criteria) were recruited at the University Psychiatry Clinics (UPK) in Basel, Switzerland between March 2017, and January 2020. All participants met the criteria for a mild depressive episode, assessed with the Hamilton Depression Rating Scale (HAMD-17 [43]) score 7 [44], and received treatment as usual (TAU) for depression (Supplementary Table 1). Excluded were patients with comorbid psychiatric disorders, with current medical conditions, with dietary restrictions and with a body mass index (BMI) > 30. Additionally, immunosuppressed and pregnant or breast-feeding patients were excluded. For more details on inclusion and exclusion criteria see [9]. All participants provided written informed consent prior to the initiation of the study, and the study was approved by the local ethics committee (Ethikkommission Nordwest- und Zentralschweiz).

### Study intervention

Participants received either a placebo or a probiotic supplement in addition to TAU over four weeks. The probiotic supplement (DSFormulation; Vivomixx®; Visbiome®) consisted of eight different bacterial strains: *Streptococcus thermophilus* NCIMB 30438, *Bifidobacterium breve* NCIMB 30441, *B. longum* NCIMB 30435 (Re-classified as *B. lactis*), *B. infantis* NCIMB 30436 (Re-classified as *B. lactis*), *Lactobacillus acidophilus* NCIMB 30442, *L. plantarum* NCIMB 30437, *L. paracasei* NCIMB 30439, and *L. delbrueckii subsp. bulgaricus* NCIMB 30440 (Re-classified as *L. helveticus*). The daily dose consisted of two sachets containing a high dose of 900 billion colony forming units (CFU)/day that could be mixed with any cold, non-carbonated drink. In the control group, participants received a placebo containing maltose and no bacteria which was indistinguishable in color, shape, size, smell, and taste from the probiotic supplement.

### Study design and procedure

Participants were randomly allocated to one of the two study arms and assessed at three time points: Week zero (baseline), week four (post-intervention) and week eight (follow-up). A standardized clinical assessment of depression (HAMD-17, Beck Depression Inventory-II (BDI-II) [45]) was conducted at all three time points. Blood samples and subjective measurements of appetite were obtained at all three time points, and saliva samples at baseline and post-intervention (see Supplementary Fig. 1). During the intervention period, all medication of the participants was registered (see Supplement) and a standardized diet containing stable amounts of fibers, starch and protein was provided. Fidelity to the protocol was assessed by the nursing personnel administering the intervention.

### Blood analysis of immune-inflammatory markers and gut hormones

Blood serum concentrations of immune-inflammatory markers (IL-1β, IL-6, macrophage inhibitory factor (MIF), and CRP) and gut-related hormones (ghrelin and leptin) were obtained. Blood samples were collected at 7 am after overnight fasting according to a standardized laboratory procedure using a serum Monovette® (Sarstedt; Nümbrecht, Germany) per manufacturer’s protocol and stored at −80 °C up until further analysis. Analysis of immune-inflammatory markers and gut-related hormones was performed by an external laboratory (Labor Rothen AG, Basel, Switzerland). Quantitative CRP was determined in the laboratory using the CRP Latex reagent system on Beckman Coulter AU Analyzers, while the other immune-inflammatory markers and gut-related hormones were measured by the U-Plex® Metabolic Group 1 (Human) Multiplex Assays by Meso Scale Discovery®. Ghrelin was assessed as total ghrelin, containing both forms acylated (active) and desacylated (inactive) ghrelin.

### Saliva cortisol analysis

The saliva concentration of the stress-hormone cortisol was obtained. Saliva samples were drawn at 9 pm before going to bed, and at the following morning at 7 am immediately upon awakening (S1), and after 10 min (S2), 20 min (S3), and 30 min (S4). A blue cap Salivette® (Sarstedt; Nümbrecht, Germany) with synthetic swab was employed to obtain the cortisol concentrations. Saliva samples were kept frozen at −80 °C until analysis. A time-resolved fluorescence immunoassay was used to determine cortisol concentrations by the biochemical laboratory from the Department of Biological and Clinical Psychology at the University of Trier, Germany. The cortisol awakening response (CAR) of participants was computed as the area under the curve with respect to the cortisol increase (AUCi) of the morning cortisol concentrations S1 to S4 [46].

### Analysis of subjective appetite ratings

Subjective measures of appetite-related sensations were assessed using a 10-point Likert Scale in the morning after overnight fasting in addition to the blood and saliva sampling. The Likert scale was anchored by two contrasting descriptors, “not at all” and “extremely” accompanied by four measures of appetite-related sensations (“hunger”, “desire to eat”, “feeling of fullness”, “satiety”).

### Transcriptomic analysis

Blood samples were collected at 7 am after overnight fasting according to a standardized laboratory procedure into a PAXgene tube (Qiagen; Hilden, Germany) per manufacturer’s protocol and stored at −80 °C up until further analysis. RNA isolation (Quantification-OD measurement, Gel electrophoresis-integrity, RNA isolation PaxGene) has been conducted by Qiagen (Qiagen; Hilden, Germany). RNA sequencing and quantification was performed at the Lausanne Genomic Technology Facility. Differential gene expression analysis (DGE) and weighted gene co-expression network analysis was performed at the Swiss Institute of Bioinformatics (SIB), University of Lausanne. Detailed descriptions of all procedures are provided in the supplement.

### Statistical analysis

All analyses were conducted on a modified intention-to-treat (mITT) sample excluding non-compliant participants and drop-outs. The compliance rate cut-off of > 65% resulted in the exclusion of two patients per group from the study sample (for details see [9]). All analyses and visualizations were performed in R (v4.3.1). Unless otherwise specified, the significance level was set at p < 0.05, and multiple comparison adjustments were performed, which were selected based on variance equality and assessed outcome variable. Additional analysis details are provided in the supplement.

### Effect of probiotics on blood/saliva markers and appetite measures

For blood and saliva measures, a Tukey transformation [47] was performed to reach quasi-normal distributions, and outlier values (defined at ±1.5 x interquartile range) were excluded (sensitivity analyses on the findings including all samples are provided in the supplement). Linear mixed-effects models (LMM) were applied to assess the probiotic effect including the following design formula: measure ~ group x time + age + sex + BMI + (1|ID), with group as a two-levelled factor (probiotics, placebo) and time as a three-levelled factor (baseline, post-intervention, follow-up). LMM included a random effect for participant, to account for individual differences. To avoid confounding, sex, age, and body-mass-index (BMI), were additionally added as fixed effects in the model. An analysis of variance (ANOVA, type III) was computed, and for significant main effects of group, time, and group x time interactions pairwise post-hoc multiple comparisons using estimated marginal means with t-tests were performed. Multiple comparison adjustments for post-hoc contrasts of equal variances were made using the Bonferroni method.

### Association between probiotics’ effect on blood/saliva markers and depression

Partial correlation analyses were conducted for both treatment groups, to explore associations between significant probiotic-induced changes on blood/saliva markers and depressive symptoms (HAMD-17). Age, sex, and BMI were included as covariates. Fischer’s z test was applied to compare correlations between treatment groups. Cook’s Distance, with a cutoff of >4/N, was used for bivariate outlier detection (sensitivity analyses on the findings including all samples are provided in the supplement).

### Effect of probiotics on gene expression and functional enrichment analysis

For gene expression measures, a negative binomial generalized linear model with the following design formula: expression ~ group + group x participant + group x time, was applied. Differentially expressed genes (DEGs) were identified using Wald’s test p < 0.05 and |fold-change| > 1.5. REACTOME gene set enrichment analysis (GSEA) was performed on the entire lists of expressed genes pre-ranked by signed p-value as determined by Wald’s test. Benjamini-Hochberg corrections were applied to functional enrichment p values to correct for multiple comparisons in high-dimensional data.

### Association between probiotics’ effect on biological/ clinical measures and DEGs

To evaluate the link between transcriptional changes and the effect of probiotics on biological and clinical measures, LMMs were applied including gene expression as fixed effect. The LMM was built and analyzed analog to the description of LMM analysis in the previous section, with the addition of a three-way interaction group x time x gene expression. Only DEGs (log transformed and normalized gene counts) significantly different between the groups over time were analyzed. The same analysis was extended to gene modules from the WGCNA by replacing individual gene counts with “module eigengene” (ME) as fixed effects.

## Results

### Participant characteristics

The final study sample included 43 participants (mITT; see [9] for details). Blood samples of N = 40 (93%), saliva samples of N = 38 (88%), transcriptome data of N = 35 (81%), and appetite measures of N = 43 (100%) were available (Supplementary Figure 1). Baseline characteristics of all participants are presented in Table 1. Group comparisons showed no significant differences in demographic characteristics, medication, and clinical measures at baseline except for HAMD-17 scores, which showed a higher score for the probiotics group (W = 311, p < 0.05). Blood, saliva, and appetite measures showed no differences between the study groups at baseline except for MIF, which showed a lower concentration for the probiotics group (W = 250, p < 0.05).

**Table 1:** Demographics, clinical characteristics, and secondary measures at baseline.

	Probiotics group	Placebo group	Group comparison
Demographic	**(n** = **19)**	**(n** = **24)**	
Age	39.21 (11.53)	38.04 (10.24)	W = 238.5, *p* = *0.81*
Sex, n (%; female)	14 (74)	12 (50)	χ^2^(1) = 1.60, *p* = *0.21*
BMI	23.83 (3.66)	25.13 (4.01)	W = 177, *p* = *0.30*
Compliance rate	87 (8.44)	88 (8.17)	W = 186, *p* = *0.84*
Depression severity			
HAMD-17	19.13 (4.89)	16.5 (4.18)	W = 311, *p* = *0.04*
BDI-II	21.53 (7.59)	22.31 (9.94)	W = 218.5, *p* = *0.96*
Medication (DDD)			
Antidepressant equivalents	1.86 (1.30)	1.82 (1.12)	W = 227, *p* = *0.99*
Antipsychotic equivalents	0.33 (0.71)	0.24 (0.31)	W = 241, *p* = *0.76*
Clinical measures			
N of hospitalizations	2.29 (1.48)	1.85 (1.23)	W = 210.5, *p* = *0.32*
STAI 1	49 (14.11)	51.83 (10.61)	W = 191, *p* = *0.68*
GSRS	28.16 (9.65)	29.96 (12.79)	W = 211.5, *p* = *0.87*
**Blood measures**			
Immune-inflammatory markers	**(n** = **18)**	**(n** = **22)**	
IL-1β^a^	0.002 (0.003)	0.001 (0.001)	W = 73.5, *p* = 0.49
IL-6^a^	0.098 (0.076)	0.104 (0.081)	W = 166, *p* = 0.86
MIF^a^	169.15 (88.84)	251.19 (106.21)	W = 250, *p* = 0.01
CRP^b^	1.78 (1.36)	1.11 (0.82)	W = 110, *p* = 0.08
Gut-related hormones			
Ghrelin^a^	7.92 (6.26)	8.43 (5.99)	W = 191, *p* = 0.72
Leptin^b^	0.014 (0.013)	0.01 (0.011)	W = 103, *p* = 0.24
**Saliva measures**			
Cortisol	**(n** = **17)**	**(n** = **21)**	
Evening cortisol^c^	1.03 (1.09)	1 (0.37)	W = 136, *p* = 0.80
Waking cortisol^c^	6.55 (3.72)	8.05 (4.36)	W = 120, *p* = 0.41
CAR^c^	7.09 (6.86)	7.99 (5.71)	W = 145, *p* = 0.99
Appetite measures	**(n** = **19)**	**(n** = **24)**	
Satiety	4.76 (2.73)	4.55 (2.72)	W = 164, *p* = 0.87
Hunger	4.29 (2.28)	5 (2.64)	W = 193.5, *p* = 0.48
Feeling of fullness	2.35 (1.93)	3.35 (2.62)	W = 207, *p* = 0.23
Desire to eat	3.82 (2.65)	5.05 (2.91)	W = 212.5, *p* = 0.20

Measures are presented as mean (SD) unsless stated otherwise. *BMI* Body mass index, *HAMD-17* Hamilton Rating Scale for Depression 17-item, *BDI-II* Beck Depression Inventory 2nd edition, *DDD* Defined Daily Dose, *STAI 1* State-Trait Anxiety Inventory 1, *GSRS* Gastrointestinal Symptom Rating Scale, *IL* Interleukin, *MIF* Macrophage Inhibitory Factor, *CRP* C-reactive Protein, *CAR* Cortisol Awakening Response. ^a^in pg/ml, ^b^in mg/l, ^c^in nmol/l.

### Blood analysis

#### Immune-inflammatory markers

**Fig. 1:**
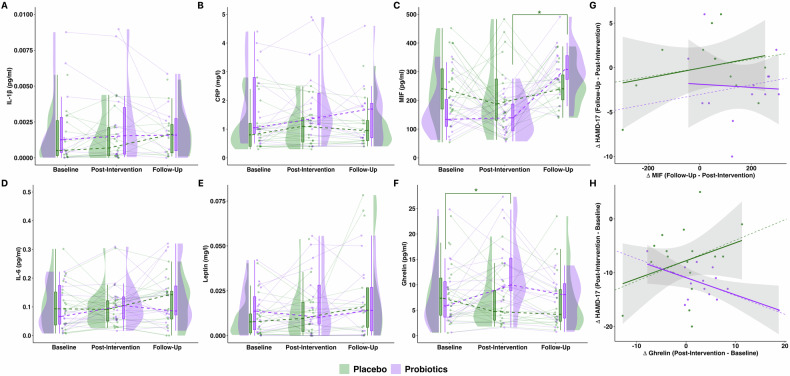
Blood concentration levels of IL-1β, CRP, MIF, IL-6, Leptin, Ghrelin in the probiotics and placebo groups from baseline to post-intervention (week 4) and follow-up assessment (week 8), and the association between changes in Ghrelin and MIF concentrations to changes in HAMD-17 scores. Concentration levels in **A**–**F** are presented as boxplots of Median [IQR], association in **G** and **H** are presented as Pearson’s correlation (solid line) and partial correlation (dotted line) controlled for (age, sex, BMI). In **A**–**F** significant group × time interactions are depicted (*p ≤ 0.05). **A** Trajectory of IL-1β blood concentration levels throughout the study. **B** Trajectory of CRP concentration levels throughout the study. **C** Trajectory of MIF concentration levels throughout the study, indicative of a significant increase from post-intervention to follow-up in the probiotics group. **D** Trajectory of IL-6 concentration levels throughout the study. **E** Trajectory of leptin concentration levels throughout the study. **F** Trajectory of ghrelin concentration levels throughout the study, indicative of a significant increase from baseline to post-intervention in the probiotics group. **G** Association between changes in MIF concentration levels and HAMD-17 scores from post-intervention to follow-up for both study group. Placebo group (Pearson’s correlation: r = 0.27, p = 0.4; partial correlation: r = 0.23, p = 0.55). Probiotics group (Pearson’s correlation: r = −0.06, p = 0.86; partial correlation: r = 0.17, p = 0.64). **H** Association between changes in ghrelin concentration levels and HAMD-17 scores from baseline to post-intervention for both study group. Placebo group (Pearson’s correlation: r = 0.3, p = 0.22; partial correlation: r = 0.35, p = 0.20). Probiotics group (correlation: r = −0.58, p = 0.03; partial correlation: r = −0.63, p = 0.04).

**Fig. 2:**
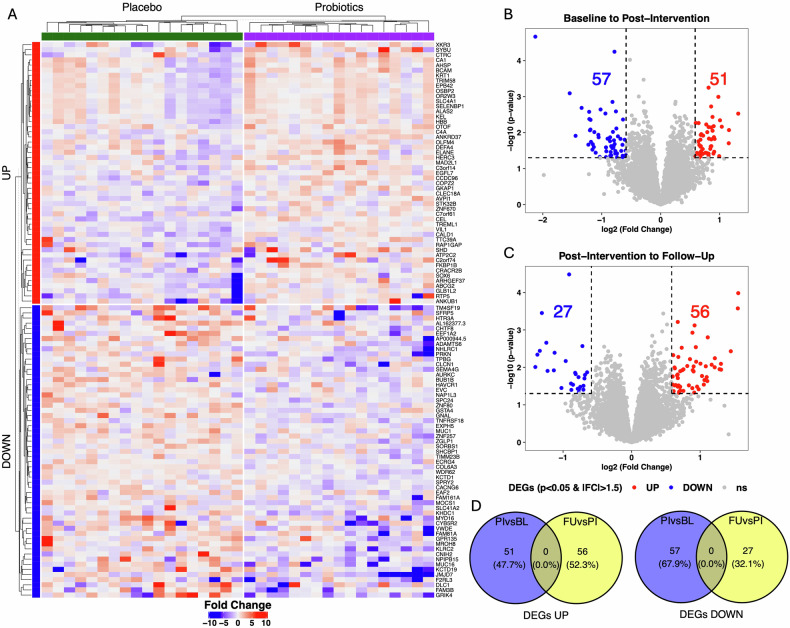
Gene expression changes from baseline to post-intervention (week 4) and follow-up assessment (week 8) in the probiotics compared to placebo groups. Differentially expressed genes (DEGs) were defined as |fold-change| > 1.5 and p value < 0.05. **A** Heatmap of differential gene expression (DGE) from baseline to post-intervention assessment in the probiotics compared to placebo groups. Genes and participants are clustered based on Euclidean distance using complete linkage method. Participants are additionally split according to their treatment groups. Genes are additionally split according to their increasing (UP) or decreasing (DOWN) expression. **B** DEGs in the probiotics compared to placebo group between the timepoints baseline to post-intervention. **C** DEGs in the probiotics compared to placebo group between the timepoints post-intervention to follow-up. **D** Overlap of UP and DOWN DEGs in the probiotics compared to placebo groups between the study periods baseline to post-intervention (PIvsBL) and post-intervention to follow-up (FUvsPI).

IL-1β and IL-6 levels did not show a significant main effect of group or time, nor a significant group x time interaction (Supplementary Table 2, Fig. 2A, D). MIF levels did not show a main effect of group, but a significant main effect of time (F(2, 96) = 11.67, p < 0.001), and a significant group x time interaction (F(2, 96) = 4.66, p < 0.05) was observed (Supplementary Table 2, Fig. 1C). Post-hoc tests demonstrated a significant increase in MIF levels from baseline to follow-up (p_Bonferroni_ < 0.01) and from post-intervention to follow-up (p_Bonferroni_ < 0.001) in the probiotics group, but not from baseline to post-intervention. No change was observed in the placebo group at any time point. CRP levels showed a main effect of group (F(1, 34.75) = 9.57, p < 0.01), but no main effect of time or group x time interaction was observed (Supplementary Table 2, Fig. 2B). Post-hoc tests demonstrated higher CRP levels across all time points in the probiotics compared to the placebo group (p_Bonferroni_ < 0.01).

#### Association between probiotics’ effect on MIF and depression symptoms

The partial correlation between MIF and HAMD-17 change-scores (x_Follow-Up_ − x_Post-Intervention_) did not demonstrate associations in the probiotics group (r = 0.17, p = 0.64) nor in the placebo group (r = 0.23, p = 0.55) (Fig. 1G). The obtained correlations did not significantly differ between the groups (z = 0.14, p = 0.89).

#### Gut-related hormones

Ghrelin levels did not show a main effect of group or time, but a significant group x time interaction (F(2, 64.69) = 4.36, p < 0.05) was observed (Supplementary Table 3, Fig. 1F). Post-hoc tests demonstrated a significant increase of ghrelin levels from baseline to post-intervention (p_Bonferroni_ < 0.05) in the probiotics group, which was no longer observable at follow-up. No change was observed in the placebo group at any time point.

Leptin levels showed a significant main effect of time (F(2, 58.77) = 4.18, p < 0.05), but no main effect of group and no group x time interaction was observed (Supplementary Table 3, Fig. 1E). Post-hoc tests demonstrated an increase of leptin levels from baseline to follow-up (p_Bonferroni_ < 0.05) across both groups.

#### Association between probiotics’ effect on ghrelin and depression symptoms

The partial correlation between ghrelin and HAMD-17 change-scores (x_Post-intervention_ − x_Baseline_) demonstrated a significant negative correlation in the probiotics group (r = −0.63, p < 0.05) but not in the placebo group (r = 0.35, p = 0.2) (Fig. 1H). The obtained correlations did significantly differ between the groups (z = 2.76, p < 0.01). That is the higher the increase in ghrelin levels from baseline to post-intervention the stronger the decrease in HAMD-17 for the probiotics but not the placebo group.

### Saliva cortisol

Evening cortisol concentrations (9 pm), waking cortisol concentrations (7 am) and the CAR did not show a significant main effect of group or time, nor a significant group x time interaction (Supplementary Table 4, Supplementary Fig. 2).

### Subjective appetite ratings

The appetite sensations, hunger, satiety, fullness, and desire to eat did not show a significant main effect of group or time, nor a significant group x time interaction (Supplementary Table 5, Supplementary Fig. 3). The partial correlation between ghrelin and subjective appetite ratings change-scores (x_Post-intervention_ − x_Baseline_) demonstrated for the feeling of fullness a significant positive correlation in the probiotics group (r = 0.61, p < 0.05) but not in the placebo group (r = 0.36, p = 0.31) (Supplementary Table 6). The obtained correlations did not significantly differ between the groups (z = 1.24, p = 0.21), indicating that the association between higher ghrelin levels and increased feelings of fullness observed in the probiotics group is not unique to the intervention. All additional partial correlations between changes in ghrelin and subjective appetite ratings did not demonstrate significant results for both groups (Supplementary Table 6).

### Transcriptomics analysis

#### DGE and functional enrichment

**Fig. 3:**
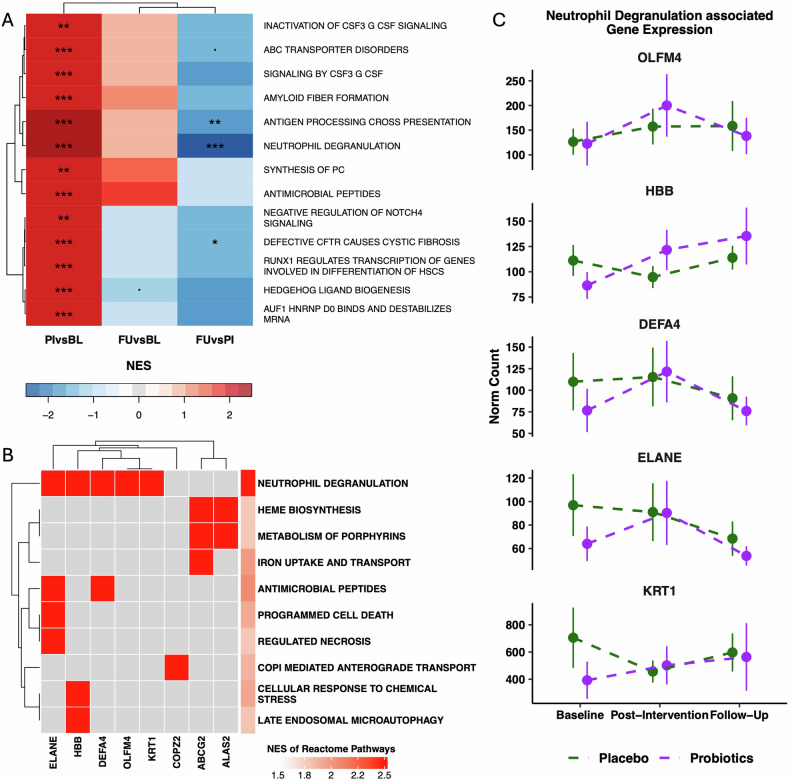
Gene set enrichment analysis of DGE associated with probiotics compared to the placebo groups. **A** Comparison of enrichment profiles of REACTOME pathways between the baseline (BL), post-intervention (week 4; PI), and follow-up (week 8; FU) assessment. Significantly upregulated pathways between PI and BL, in probiotics compared to placebo groups were filtered for display (adjusted p ≤ 0.01 and |NES| > 2). Color of cells indicates the NES. Corresponding BH-adjusted p values are also indicated (*p ≤ 0.05, **p ≤ 0.01, ***p ≤ 0.001). **B** Core enriched DEGs for GSEA enriched REACTOME pathways at post-intervention vs baseline. **C** Trajectories of norm count of core enriched DEGs throughout the study period associated to the pathway neutrophil degranulation. Norm counts are present as M (SE), axes have been edited according to respective norm count for clearer interpretation.

**Fig. 4:**
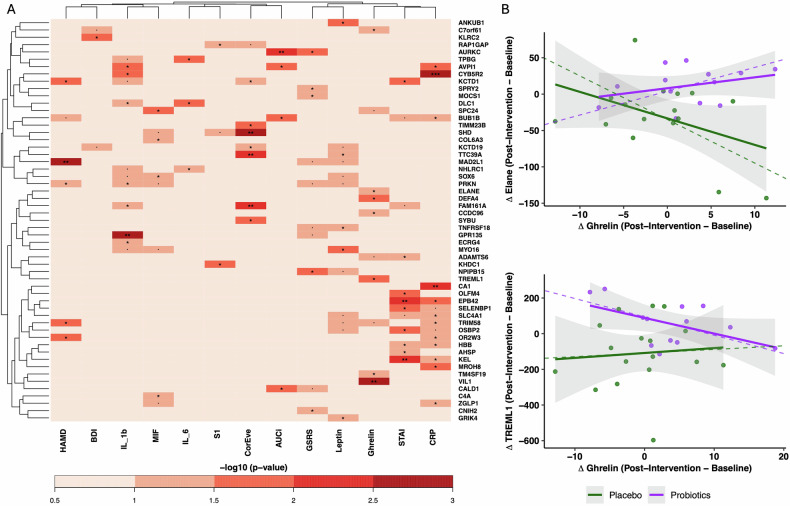
Association between biological/clinical measures and probiotics-induced DEGs from baseline to post-intervention assessment (week 4). **A** Effect of group x time x gene expression interaction on the biological/clinical measures assessed by LMM. The color scale represents -log scaled ANOVA p-values. Significant interactions are depicted (*p ≤ 0.05, **p ≤ 0.01, ***p ≤ 0.001). **B** Association between the change of ghrelin and significantly associated genes (*p ≤ 0.05) from baseline to post-intervention for both study group. Only DEGs with a significant association to ghrelin are presented. Associations were assessed as Pearson’s correlation (solid line) and partial correlation (dotted line) controlled for (age, sex, BMI). ELANE: Placebo group (correlation: r = −0.42, p = 0.1; partial correlation: r = −0.69, p = 0.01). Probiotics group (correlation: r = 0.32, p = 0.28; partial correlation: r = 0.7, p = 0.02). TREML1: Placebo group (correlation: r = 0.08, p = 0.75; partial correlation: r = 0.061, p = 0.83). Probiotics group (correlation: r = −0.51, p = 0.07; partial correlation: r = −0.6, p = 0.07).

From baseline to post-intervention, DGE analysis revealed the upregulation of 51 genes (fold change > 1.5) and downregulation of 57 genes (fold change < −1.5) (unadjusted p < 0.05) in the probiotics compared to the placebo group (Fig. 2A, B). Gene set enrichment analysis (GSEA) of REACTOME pathways demonstrated the DGE to be a coordinated upregulation of genes involved in functional pathways of immune activation. Probiotic supplementation was associated with an upregulation of biological processes of “Neutrophil degranulation”, “Antigen processing cross presentation”, “Signaling by CSF3 (G-CSF)”, “Antimicrobial peptides”, “Negative regulation of NOTCH4 signaling”, “Inactivation of CSF3 (G-CSF) signaling”, all functionally associated to immune mechanisms (Fig. 3A). Analyses of overlapping DEG and enriched pathways identified the DEG (HBB, ELANE, DEFA4, OLFM4, KRT1) in “Neutrophil degranulation”, and the DEG (ELANE, DEFA4) in “Antimicrobial peptides” (Fig. 3B, C). The GSEA of downregulated genes revealed no functional coordination, as no association to consensus REACTOME pathways was obtained. The DGE from post-intervention to follow-up demonstrated no upregulation of genes functionally associated to immune activity, indicating a transient and immediate effect of probiotic supplementation on DGE related to immune activation (Fig. 3A). Changes in multiple DEGs were significantly linked to changes in ghrelin levels in the probiotics compared to the placebo groups from baseline to post-intervention (Fig. 4A). For two DEGs (TREML1 and ELANE) the change score (x_Post-intervention_ − x_Baseline_) demonstrated a significant partial correlation to the ghrelin change score (x_Post-intervention_ − x_Baseline_) in the probiotics group (Fig. 4B).

#### Weighted gene correlation network analysis

**Fig. 5:**
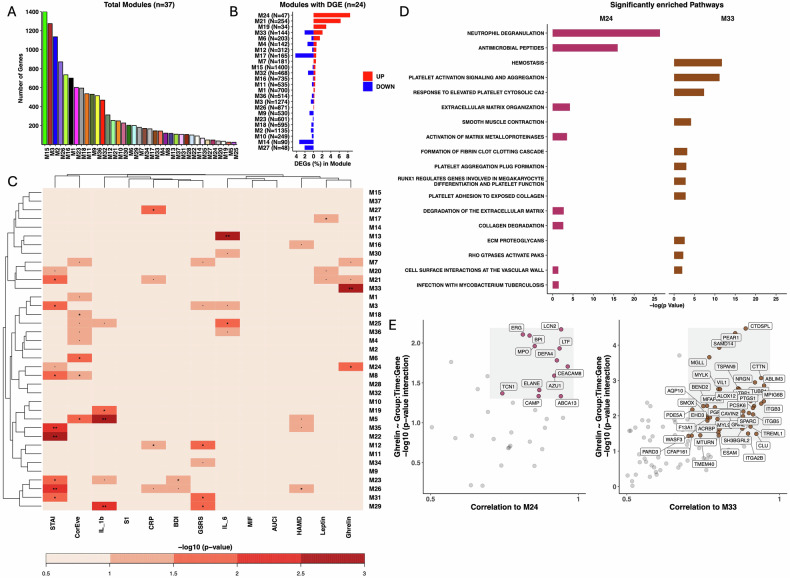
Co-expressed gene modules identification and their association to the probiotics intervention and changes in biological/clinical measures. **A** Identification of co-expressed gene modules based on a weighted gene co-expression network analysis. Colors are randomly assigned to facilitate module identification. **B** Percentage of probiotics-induced DEGs in modules. N next to module indicates absolute number of DEGs in modules. **C** Effect of group × time × ME (“module eigengene”) interaction on the biological/clinical measures assessed by LMM. The color scale represents −log scaled ANOVA p-values. Significant interactions are depicted (*p ≤ 0.05, **p ≤ 0.01, ***p ≤ 0.001). **D** Significantly enriched REACTOME pathways in modules associated to ghrelin. BH-adjusted p values for the enrichment test (*p ≤ 0.05). **E** Identification of genes with a high Module Membership (correlation to ME) and association to ghrelin (LMM assessed significant group × time × gene expression) in M24 and M33.

To capture correlation pattern among genes in response to the probiotic intervention, we grouped co-expressed genes into 37 modules using a network-based approach [48]. We found that the Module 24 (M24) exhibited the highest degree of overlap to DEGs, containing 47 (8.5%) upregulated DEGs (Fig. 5B). The REACTOME over-representation analysis of module genes found M24 to be enriched for functional pathways which we already identified as upregulated in the DGE analysis (“Neutrophil degranulation” and “Antimicrobial peptides”) (Fig. 5D). This indicates that the module M24 recapitulates probiotics-specific transcriptional changes on immune activity. Interestingly, we also found that the interaction group × time × ME (“module eigengene”) of M24 was significantly associated to ghrelin (Fig. 5C, E).

## Discussion

We previously reported a beneficial impact of a multi-strain probiotic intervention on both depressive symptoms and gut microbiota composition in individuals with depression receiving TAU [9]. Following this, the current secondary analysis focusing on immune-inflammatory mechanisms of probiotics revealed three key outcomes: Firstly, probiotics exhibited a significant, albeit transient, increase in circulating levels of ghrelin over the intervention period. This effect was associated with the improvement in depressive symptoms during the intervention phase. Secondly, probiotics showed no immediate effect on pro-inflammatory cytokines, cortisol concentrations, and leptin. However, MIF levels showed a significant increase at follow-up in the probiotic group. Thirdly, probiotics induced changes in gene expression patterns functionally associated with the immune system. Similar to the elevation of circulating ghrelin, the transcriptional changes were only evident during the intervention period and no longer evident at follow-up.

The association between the probiotic-induced increase of ghrelin levels and decrease of depressive symptoms adds to the existing literature suggesting antidepressant effects of ghrelin [49, 50]. Multiple preclinical studies reported antidepressant-like properties of ghrelin in rodents [51–55]. Clinical data show a more mixed picture of ghrelin’s effect on depressive symptoms [49, 56]. There is evidence for improved depressive symptoms following ghrelin administration in a study on patients with MDD [57], while another study reported an association between higher severity of depressive symptoms and increased ghrelin concentrations in patients with depression [58]. However, other studies reported no such association in patients with depression [59–61]. In addition, compared to healthy individuals, in patients with depression higher [62–64], lower [65] and comparable [61, 66, 67] ghrelin concentrations were reported, indicating variability amongst patients with depression in terms of ghrelin concentrations. In this study, the obtained increase in ghrelin levels was due to the intervention effect of a multi-strain probiotic over four weeks, which returned to baseline concentrations at follow-up four weeks after the intervention was completed. The finding of higher ghrelin concentrations following probiotic supplementation in patients with depression is in line with preclinical results reporting higher ghrelin gene expression in mice treated with the same multi-strain probiotic [68]. Furthermore, mice treated with the probiotic were found to have increased numbers of ghrelin secreting cells in the mouse intestine, alongside higher numbers of cells shielding the gastric epithelium [68]. In addition, probiotics (*Lactobacillus* spp.) were reported to enhance ghrelin gene expression and ghrelin secretion in other animal studies [69, 70]. These findings stand in contrast to another animal study reporting reduced ghrelin concentrations after administering the same multi-strain probiotic we used [71]. However, none of these preclinical findings were obtained using an animal model of depression, limiting the translation to a clinical population of patients with depression. Nonetheless, in accordance with our initial finding of increased abundances of the genus *Lactobacillus* following the probiotic supplementations [9], most animal studies demonstrated that probiotics (*Lactobacillus* spp.) have the potential to enhance ghrelin gene expression and secretion.

We found no treatment effect over time of probiotic supplementation on all immune-inflammatory blood markers except for MIF, for which we obtained a significant increase at follow-up. The lack of effects at post-intervention is in line with other clinical trials in patients with depression, which did not find the proposed anti-inflammatory properties of probiotics reported in animal models [72, 73]. In accordance with our results, no changes in IL-1β and IL-6 levels, were reported in a human study administering the same multi-strain probiotic supplement we used and in a recent meta-analysis evaluating probiotics trials [4, 74]. However, while most results point towards no changes in immune-inflammatory markers, two trials using the same probiotic strains in different supplements reported decreased hs-CRP levels in patients with depression [8], and reduced IL-6 levels in patients with multiple sclerosis [75]. These mixed findings on probiotic supplement effects on immune-inflammatory markers might be due to the variability in used probiotic strains and targeted clinical populations [76]. Some probiotics may exhibit anti-inflammatory properties, while others might function as immune stimulants, thereby enhancing physiological inflammation [77]. In line with this, for the probiotic supplement used in our study, increased macrophage activation has been reported [78, 79]. This may explain our probiotic-induced increase in MIF concentrations at follow-up. Macrophages are known to release cytokines, including MIF, and activate the innate immune system [80]. MIF, in turn, acts as a regulator of the innate immune activity but has also been reported to play a crucial role in maintaining the barrier function of intestinal epithelial cells [81] and is associated with enhanced macrophage-dependent pathogen clearance [82]. Furthermore, previous research reported for probiotics of the genus Lactobacillus to stimulate MIF production [83]. The obtained increase in MIF concentration levels from post-intervention to follow-up suggests a delayed response in the immune system, where the effect of probiotics on MIF became more apparent only after the intervention ceased. In the context of depression, delayed or dysregulated immune responsivity is a common feature [84]. Our results, together with previous findings, propose that probiotics play a modulatory role in immune-inflammatory processes, activating both stimulatory and inhibitory responses depending on context and probiotic strains.

Gene expression analysis revealed transcriptional changes in the probiotic group compared to the placebo group during the intervention. Our study found 108 DEGs following a multi-strain probiotic intervention. Only one other study has examined gene expression changes after probiotic administration in patients with depression, reporting higher IL-6 expression post-intervention [85]. Other studies have observed anti-inflammatory gene expression changes with probiotic supplementation in neurodegenerative disorders [86, 87]. Our findings showed that DEGs were related to immune activation pathways rather than anti-inflammatory processes, aligning with the literature suggesting different probiotic strains have distinct biological mechanisms [88]. Specifically, neutrophil-associated immune activation was the most significant effect, consistent with preclinical research indicating that the same multi-strain probiotic activates the epithelial innate immune system [77]. This “physiologic inflammation” induced by probiotics is proposed to enhance defense against harmful gut bacteria and support the innate immune system. Using WGCNA, we identified module M24, which captured upregulated immune activity and was associated with probiotic-induced ghrelin changes over time. M24 includes DEGs such as Olfactomedin 4 (OLFM4), Elastase (ELANE), and Defensin Alpha 4 (DEFA4), which are important for gastrointestinal defense. OLFM4 and DEFA4, in particular, have been linked to host defense in the mature intestine [89], and increased expression following probiotics supplementation of the genus *Lactobacillus* in mice [90]. OLFM4 is part of the gastrointestinal mucosal surface and may play a role in epithelial defense [91], while DEFA4 is involved in killing Gram-negative bacteria associated with gut dysbiosis [92].

This study had several strengths and limitations that necessitate careful consideration. A key strength lies in the systematic and comprehensive examination of diverse biological parameters associated with the mechanisms of the MGB axis involved in depression. By employing a combination of transcriptomics and blood concentration analyses, our methodology facilitated an in-depth exploration of the biological underpinnings relevant to the reported antidepressant mechanisms of probiotics. Notwithstanding these strengths, several limitations should be noted. The small sample size limits the generalizability of our findings, and the analyses conducted were exploratory and not a priori powered. Consequently, for the transcriptomics results of DGE, unadjusted p-values are reported which should be interpreted with caution. Additionally, the CAR was sampled until 30 min post-awakening, while consensus guidelines suggest sampling up to 30–45 mins post-awakening [93]. Moreover, we analyzed and reported total ghrelin levels without distinguishing between the acylated and desacylated forms. Considering the contrasting roles on energy balance reported for both forms (desacylated ghrelin is proposed to be a functional inhibitor of acylated ghrelin) [94, 95], distinguishing both forms would have provided more precise insights. Especially in the context of depression, as there is no conclusive evidence yet on the link of acylated, desacylated, or total ghrelin to depression and treatment response. Lastly, despite our efforts to record and control for medication effects, we cannot definitively ascertain whether the observed effects are specific to interactions with certain antidepressants, and the inclusion criteria (HAMD-17 > 7) led to a heterogeneous sample in terms of symptom severity. Future studies with larger sample sizes are necessary to validate and potentially extend our findings to patients with moderate to severe depression. Additionally, differentiating between both forms of ghrelin is crucial in future research to better understand their respective link to probiotics and their impact on depressive symptoms.

In conclusion, the biological mechanisms of add-on multi-strain probiotic supplementation in patients with depression were linked to the gut hormone ghrelin and the upregulation of genes of immune activation. Higher ghrelin levels after probiotic supplementation were furthermore related to improved depressive symptoms, hinting at a potential link between ghrelin secretion and antidepressant mechanisms. These findings emphasize probiotics’ biological mechanism of action as promoting immune activation ultimately associated to symptom relief in patients with depression. Moving forward, our results warrant replication in large-scale mechanistic trials of probiotic supplementation to test the potential of immune-inflammatory measures as stratification and treatment response biomarkers in depression. This could pave the way for more targeted and personalized approaches in the treatment of depression.

### Supplementary information

Supplemental Material

## Data Availability

The datasets generated and analyzed during the current study are available from the corresponding author on reasonable request.
