# Supplementary material for: Examining immune-inflammatory mechanisms of probiotic supplementation in depression: secondary findings from a randomized clinical trial
Source: Transl Psychiatry. 2024 Jul 24;14:305. doi: 10.1038/s41398-024-03030-7 (PMC11269721; doi:10.1038/s41398-024-03030-7)
Supplement: Supplementary file 1 — Supplemental Material [file 41398_2024_3030_MOESM1_ESM.docx]

**Examining immune-inflammatory mechanisms of probiotic supplementation in depression: secondary findings from a randomized clinical trial**

**Supplementary information (SI)**

**Supplementary Methods**

**
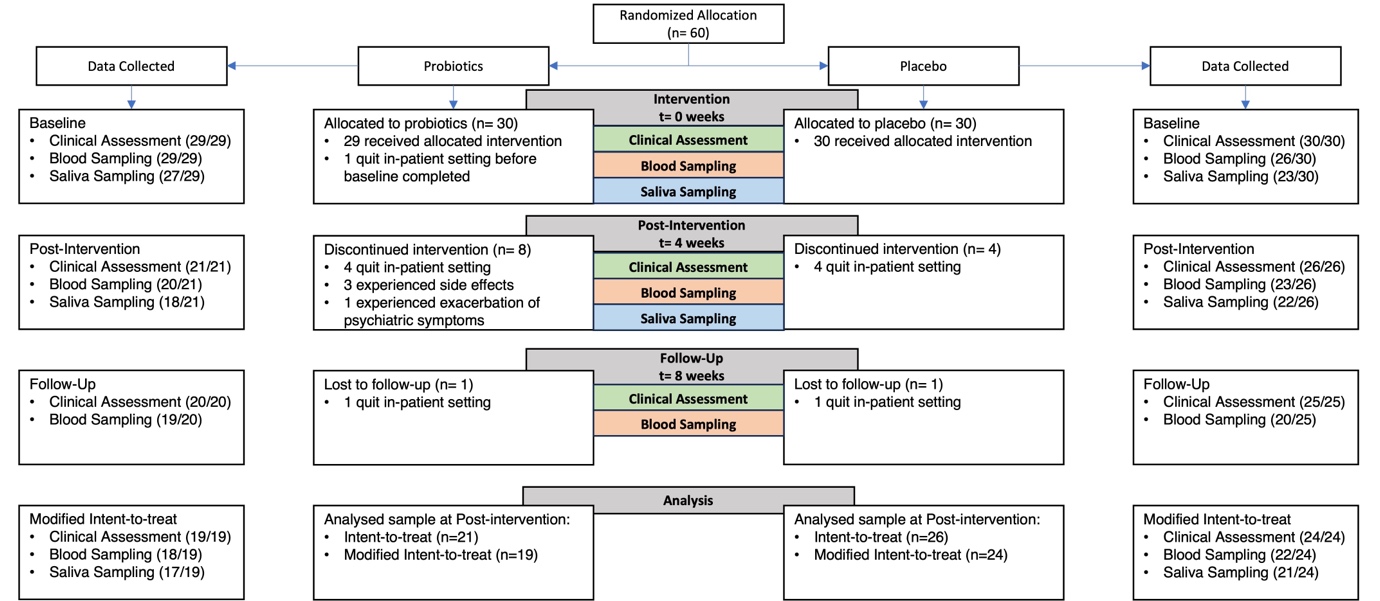
**

**Supplementary Figure 1.** CONSORT diagram on study procedure capturing all available data and performed analysis.

Treatment as Usual

To monitor the received treatment as usual, medication intake was extracted for each study participant from the clinic information system. Antidepressant and antipsychotic medications were transformed into dose equivalents based on the defined daily dose (DDD) method, which is described as the “assumed average maintenance dose per day for a drug used for its main indication in adults» ^1^. For each participant, detailed information is depicted in Table S1. Different antidepressants and antipsychotics were summarized using the DDD. Furthermore, the specific period of intake was considered as the drugs were not necessarily administered over the whole study period.

Randomization

Block randomization was performed in a 1:1 ratio by an independent researcher using a computer-based randomization algorithm to avoid systematic biases, as outlined in ^2.^ Investigators and assessors were blinded during the entire period of data collection and patients were informed about the allocation after the follow-up assessment. This was s secondary analysis of a RCT, and previous results have already been published ^2–4^. Thus, the investigators and collaborators were not blinded during data analysis.

**Supplementary Table 1.** Antidepressant and antipsychotic medication per patient in mean defined daily dose (DDD) over the four-week intervention period. Only patients that finished the intervention are included.

| **Group** | **Patient ID** | **Name** | **DDD** |
| --- | --- | --- | --- |
| Probiotics | Patient 1 | mirtazapine, fluoxetine, trimipramine, lithium, olanzapine, chlorprothixene, quetiapine | 3.45 |
|  | Patient 2 | venlafaxine, lithium, quetiapine | 0.76 |
|  | Patient 3 | venlafaxine, quetiapine | 2.33 |
|  | Patient 4 | lithium, quetiapine | 0.75 |
|  | Patient 5 | bupropion, trazodone | 1.47 |
|  | Patient 6 | venlafaxine, trazodone, quetiapine | 2.47 |
|  | Patient 7 | quetiapine | 0.01 |
|  | Patient 8 | sertraline, quetiapine | 3.22 |
|  | Patient 9 | escitalopram | 2.00 |
|  | Patient 10 | venlafaxine, quetiapine | 4.02 |
|  | Patient 11 | duloxetine, quetiapine | 1.12 |
|  | Patient 12 | lithium, citalopram, quetiapine, olanzapine | 4.33 |
|  | Patient 13 | duloxetine, quetiapine | 1.04 |
|  | Patient 14 | duloxetine | 0.50 |
|  | Patient 15 | mirtazapine, sertraline, quetiapine | 5.26 |
|  | Patient 16 | venlafaxine, quetiapine | 3.10 |
|  | Patient 17 | sertraline, mirtazapine | 3.25 |
|  | Patient 18 | bupropion, trimipramine, clotiapine, pipamperone | 0.93 |
|  | Patient 19 | duloxetine | 0.50 |
|  | Patient 20 | bupropion, quetiapine | 1.13 |
|  | Patient 21 | duloxetine | 1.00 |
| Placebo | Patient 1 | vortioxetine, quetiapine | 1.57 |
|  | Patient 2 | sertraline, vortioxetine, clotiapine | 1.91 |
|  | Patient 3 | duloxetine, olanzapine | 3.00 |
|  | Patient 4 | venlafaxine, olanzapine, clotiapine | 3.02 |
|  | Patient 5 | duloxetine, risperidone, quetiapine | 1.03 |
|  | Patient 6 | agomelatine | 1.74 |
|  | Patient 7 | venlafaxine | 2.73 |
|  | Patient 8 | mirtazapine, quetiapine | 1.06 |
|  | Patient 9 | bupropion | 1.00 |
|  | Patient 10 | bupropion | 0.45 |
|  | Patient 11 | vortioxetine | 1.00 |
|  | Patient 12 | fluoxetine, olanzapine | 1.45 |
|  | Patient 13 | escitalopram, olanzapine | 2.97 |
|  | Patient 14 | lithium, olanzapine, pipamperone | 0.77 |
|  | Patient 15 | venlafaxine, vortioxetine, olanzapine | 3.99 |
|  | Patient 16 | duloxetine | 1.55 |
|  | Patient 17 | mirtazapine | 1.50 |
|  | Patient 18 | trazodone, bupropion | 0.60 |
|  | Patient 19 | trazodone, bupropion, duloxetine | 1.30 |
|  | Patient 20 | escitalopram | 2.00 |
|  | Patient 21 | escitalopram, lithium | 4.00 |
|  | Patient 22 | venlafaxine, mirtazapine, bupropion, clotiapine | 2.61 |
|  | Patient 23 | vortioxetine, quetiapine, olanzapine, chlorprothixene | 1.54 |
|  | Patient 24 | fluoxetine, agomelatine, pipamperone, quetiapine | 5.26 |
|  | Patient 25 | vortioxetine | 2.07 |
|  | Patient 26 | vortioxetine, clotiapine, quetiapine | 2.27 |

Notes. DDD = defined daily dose.

**Transcriptomics Analysis**

RNA isolation, sequencing and quantification

RNA-seq libraries were prepared from 250 ng of total RNA with the Illumina TruSeq Stranded mRNA reagents (Illumina) and the QIAseq FastSelect -Globin reagents (Qiagen; Hilden, Germany) for globin transcript depletion. Library preparation was performed on a Sciclone liquid handling robot (PerkinElmer; Waltham, Massachusetts, USA) with a PerkinElmer-developed automated script. Unique dual indexes were used for barcoding of the libraries. Libraries were quantified by a fluorometric method (QubIT, Life Technologies) and their quality assessed on a Fragment Analyzer (Agilent Technologies). Sequencing was performed on an Illumina NovaSeq 6000 for 300 cycles (paired end 150 nt reads). Sequencing data were demultiplexed using the bcl2fastq2 Conversion Software (version 2.20, Illumina). Low quality sequences and adapters were trimmed using Cutadapt (v. 2.5) ^5^. Reads matching to ribosomal sequences were removed with fastq_screen (v. 0.11.1) ^6^ and low complexity reads were subsequently removed with reaper (v. 15-065) ^7^. Reads were aligned against Homo sapiens genome (build GRCh38 and Ensembl version 102) using STAR (v. 2.5.3a) ^8^ and read counts per gene locus were summarized using htseq-count (v. 0.9.1) ^9^.

Differential gene expression (DGE) analysis

For downstream analyses, libraries were filtered for minimal expression retaining only the genes with at least 1 count per million reads (CPM) in at least 10% of the samples.

Library size adjustment and differential gene expression analysis was done using the R package DESeq2 (v1.42.0) ^10^.

*Weighted gene correlation network analysis*

Modules of co-expressed genes were generated using a weighted gene co-expression network analysis (WGCNA) approach ^11^. WGCNA was performed on normalized, and variance-stabilizing transformed expression data measured at baseline or post-intervention from the participants who underwent transcriptional profiling. A signed hybrid network was constructed by calculating an adjacency matrix using Pearson correlation with pairwise complete observations and a soft-thresholding power of 6. A topological overlap matrix was computed from the adjacency matrix, converted to distances, and clustered by hierarchical clustering using average linkage clustering. Modules were identified by dynamic tree cut method with a minimum size of 20 and deepSplit value of 2. The expression profiles of the co-expressed genes in each module were summarized by calculating the first principal component of the corresponding expression matrix, referred to as the “module eigengene” (ME). Similar modules were merged using a ME distance of 0.2 as the threshold. MEs were also used to computationally relate modules to clinical traits. For each gene, irrespective of its original module assignment, we also defined a "module membership" (MM) by correlating its expression profile with the module eigengene of a given module.

*Statistical analysis*

To test the effect of probiotics over time, while controlling for random participant effects, we used DESeq2 to fit a negative binomial generalized linear model. REACTOME gene set enrichment analysis (GSEA) was performed on the entire lists of expressed genes pre-ranked by signed p-value as determined by Wald's test, using the "GSEA" function of the R package clusterProfiler (v4.10.0) ^12^. The enrichment scores were normalized by gene set size, and their statistical significance was assessed by permutation tests (n = 1,000). Testing for over-representation of REACTOME pathways in gene modules was performed using hypergeometric test implemented in the "enricher" function from clusterProfiler. Genes with low module membership (|MM|≤0.6) were discarded from this analysis.

**Supplementary results**

Sensitivity Analysis

Sensitivity analyses were performed to evaluate the robustness of our findings with respect to outlier detection and exclusion. Re-analysis of the linear mixed effect models (LMMs) including all samples violated the assumption of normality of residuals (Shapiro-Wilk test, p < .05) for IL-1β, IL-6, CRP and Ghrelin. This underscores the necessity of outlier detection and removal to maintain valid statistical assumptions. Additional sensitivity analyses with more stringent outlier criteria were obtained to assess the robustness of the effects. The usage of extreme outliers as exclusion criteria (extreme outliers > 3*IQR) confirmed all initial findings and maintained the assumption of normality of residuals (Shapiro-Wilk test, p > .05). For Leptin and MIF, the re-analysis including all samples confirmed the reported effects. Cortisol measures and subjective appetite ratings remained consistent when assessed with the full sample. Additionally, the association between the change in blood measures (Ghrelin and MIF) and depression symptoms (HAMD-17), was confirmed in the re-analysis using the full sample.

**Supplementary Table 2.** Concentration of inflammation markers at each time point and results of LMMs.

| **Measure** | | **Probiotic**  **(n=18)** | **Placebo**  **(n=22)** | **ANOVA*** | | | | |
| --- | --- | --- | --- | --- | --- | --- | --- | --- |
|  |  |  |  | **Effect** | **df** | **MS** | **F** | **p** |
| IL-1β^a^ | Baseline | 0.002 (0.003) | 0.001 (0.002) | Group | 1 | 0.00 | 0.23 | 0.96 |
|  | Post-Intervention | 0.002 (0.003) | 0.001 (0.002) | Time | 2 | 0.00 | 0.23 | 0.27 |
|  | Follow-Up | 0.002 (0.002) | 0.002 (0.002) | Group*Time | 2 | 0.00 | 0.57 | 0.57 |
| IL-6^a^ | Baseline | 0.1 (0.08) | 0.1 (0.08) | Group | 1 | 0.00 | 0.24 | 0.63 |
|  | Post-Intervention | 0.11 (0.07) | 0.1 (0.07) | Time | 2 | 0.01 | 1.08 | 0.34 |
|  | Follow-Up | 0.13 (0.1) | 0.12 (0.08) | Group*Time | 2 | 0.01 | 0.77 | 0.47 |
| MIF^a^ | Baseline | 169.15 (106.21) | 251.19 (88.84) | Group | 1 | 2.91 | 1.31 | 0.25 |
|  | Post-Intervention | 145.2 (64.28) | 215.1 (126.58) | Time | 2 | 25.85 | 11.67 | **0.00** |
|  | Follow-Up | 306.35 (87.91) | 253.16 (78.77) | Group*Time | 2 | 10.32 | 4.66 | **0.01** |
| CRP^b^ | Baseline | 1.78 (1.36) | 1.11 (0.82) | Group | 1 | 0.02 | 9.57 | **0.00** |
|  | Post-Intervention | 1.93 (1.4) | 1.1 (0.68) | Time | 2 | 0.00 | 1.07 | 0.35 |
|  | Follow-Up | 1.54 (1.15) | 1.16 (0.79) | Group*Time | 2 | 0.00 | 0.25 | 0.78 |

Measures are presented as mean (SD); IL-1β = Interleukin 1β; IL-6 = Interleukin 6; MIF = Macrophage Inhibitory Factor; CRP = C-reactive protein; ^a^ in pg/ml, ^b^ in mg/l; *ANOVA estimates are based on Tukey transformed values; df = degrees of freedom; MS = mean squares; p value < 0.05 significant.

**Supplementary Table 3.** Concentration of gut-related hormones at each time point and results of LMMs.

| **Measure** | | **Probiotic**  **(n=18)** | **Placebo**  **(n=22)** | **ANOVA*** | | | | |
| --- | --- | --- | --- | --- | --- | --- | --- | --- |
|  |  |  |  | **Effect** | **df** | **MS** | **F** | **p** |
| Ghrelin^a^ | Baseline | 7.92 (6.26) | 8.43 (6) | Group | 1 | 0.01 | 0.14 | 0.71 |
|  | Post-Intervention | 12.5 (7.28) | 6.7 (4.99) | Time | 2 | 0.1 | 2.86 | 0.06 |
|  | Follow-Up | 7.47 (4.24) | 7.14 (6.35) | Group*Time | 2 | 0.16 | 4.36 | **0.02** |
| Leptin^b^ | Baseline | 0.014 (0.013) | 0.01 (0.011) | Group | 1 | 0.58 | 0.24 | 0.45 |
|  | Post-Intervention | 0.018 (0.018) | 0.013 (0.015) | Time | 2 | 0.02 | 4.18 | **0.02** |
|  | Follow-Up | 0.018 (0.018) | 0.032(0.034) | Group*Time | 2 | 0.00 | 1.12 | 0.33 |

Measures are presented as mean (SD); ^a^ in pg/ml, ^b^ in mg/l; *ANOVA estimates are based on Tukey transformed values; df = degrees of freedom; MS = mean squares; p value < 0.05 significant.

**Supplementary Table 4.** Concentration of cortisol at each time point and results of LMMs.

| **Measure** | | **Probiotic**  **(n=17)** | **Placebo**  **(n=21)** | **ANOVA*** | | | | |
| --- | --- | --- | --- | --- | --- | --- | --- | --- |
|  |  |  |  | **Effect** | **df** | **MS** | **F** | **p** |
| Evening | Baseline | 1.03 (0.49) | 1 (0.37) | Group | 1 | 0.00 | 0.05 | 0.82 |
| Cortisol^a^ | Post-Intervention | 1.05 (0.67) | 1.02 (0.55) | Time | 1 | 0.00 | 0.00 | 0.97 |
|  |  |  |  | Group*Time | 1 | 0.00 | 0.00 | 0.97 |
| Waking | Baseline | 8.05 (4.36) | 6.55 (3.72) | Group | 1 | 0.12 | 1.05 | 0.31 |
| Cortisol^a^ | Post-Intervention | 6.41 (4.33) | 6.91 (4.91) | Time | 1 | 0.15 | 1.31 | 0.26 |
|  |  |  |  | Group*Time | 1 | 0.1 | 0.89 | 0.35 |
| CAR^a^ | Baseline | 7.09 (6.86) | 7.99 (5.71) | Group | 1 | 7.86 | 0.17 | 0.68 |
|  | Post-Intervention | 9.3 (8.64) | 6.85 (7.47) | Time | 1 | 5.72 | 0.13 | 0.72 |
|  |  |  |  | Group*Time | 1 | 57.28 | 1.26 | 0.27 |

Measures are presented as mean (SD); CAR = Cortisol Awakening Response; ^a^ in nmol/l; *ANOVA estimates are based on Tukey transformed values; df = degrees of freedom; MS = mean squares; p value < 0.05 significant.

**Supplementary Table 5.** Appetite measures at each time point and results of LMMs.

| **Measure** | | **Probiotic**  **(n=19)** | **Placebo**  **(n=24)** | **ANOVA** | | | | |
| --- | --- | --- | --- | --- | --- | --- | --- | --- |
|  |  |  |  | **Effect** | **df** | **MS** | **F** | **p** |
| Satiety | Baseline | 4.76 (2.73) | 4.55 (2.72) | Group | 1 | 0.00 | 0.23 | 1 |
|  | Post-Intervention | 3.81 (2.01) | 3.83 (2.48) | Time | 2 | 0.42 | 1.64 | 0.2 |
|  | Follow-Up | 3.75 (1.91) | 3.92 (2.9) | Group*Time | 2 | 0.02 | 0.07 | 0.93 |
| Hunger | Baseline | 4.29 (2.28) | 5 (2.64) | Group | 1 | 0.96 | 1.63 | 0.21 |
|  | Post-Intervention | 4.38 (2) | 5.17 (2.53) | Time | 2 | 1.24 | 2.1 | 0.13 |
|  | Follow-Up | 4.58 (1.68) | 6.31 (2.84) | Group*Time | 2 | 0.73 | 1.24 | 0.3 |
| Felling of | Baseline | 2.35 (1.93) | 3.35 (2.62) | Group | 1 | 0.03 | 0.52 | 0.48 |
| fullness | Post-Intervention | 2.31 (2.02) | 2.66 (1.88) | Time | 2 | 0.12 | 1.9 | 0.16 |
|  | Follow-Up | 3.75 (2.6) | 2.92 (2.47) | Group*Time | 2 | 0.06 | 0.97 | 0.38 |
| Desire to | Baseline | 3.82 (2.65) | 5.05 (2.91) | Group | 1 | 1.19 | 1.67 | 0.2 |
| eat | Post-Intervention | 3.44 (2.61) | 5.11 (2.74) | Time | 2 | 0.27 | 0.39 | 0.68 |
|  | Follow-Up | 3.92 (1.88) | 4.92 (3.4) | Group*Time | 2 | 0.23 | 0.33 | 0.72 |

Measures are presented as mean (SD); df = degrees of freedom; MS = mean squares; p value < 0.05 significant.

**Supplementary Table 6.** Partial correlations between the change score of ghrelin concentration levels and subjective appetite ratings per group.

|  | Group | Baseline - Post-intervention | |
| --- | --- | --- | --- |
|  |  | r | p |
| Ghrelin x Hunger | Probiotics | -0.06 | .89 |
|  | Placebo | 0.14 | .69 |
| Ghrelin x Satiety | Probiotics | -0.47 | .17 |
|  | Placebo | 0.52 | .10 |
| Ghrelin x Feeling of Fullness | Probiotics | 0.61 | .05* |
|  | Placebo | 0.36 | .31 |
| Ghrelin x Desire to eat | Probiotics | 0.10 | .81 |
|  | Placebo | 0.19 | .57 |

Partial correlations were calculated including age, sex and BMI; *p-value < .05 significant.

**Supplementary Figures**

**
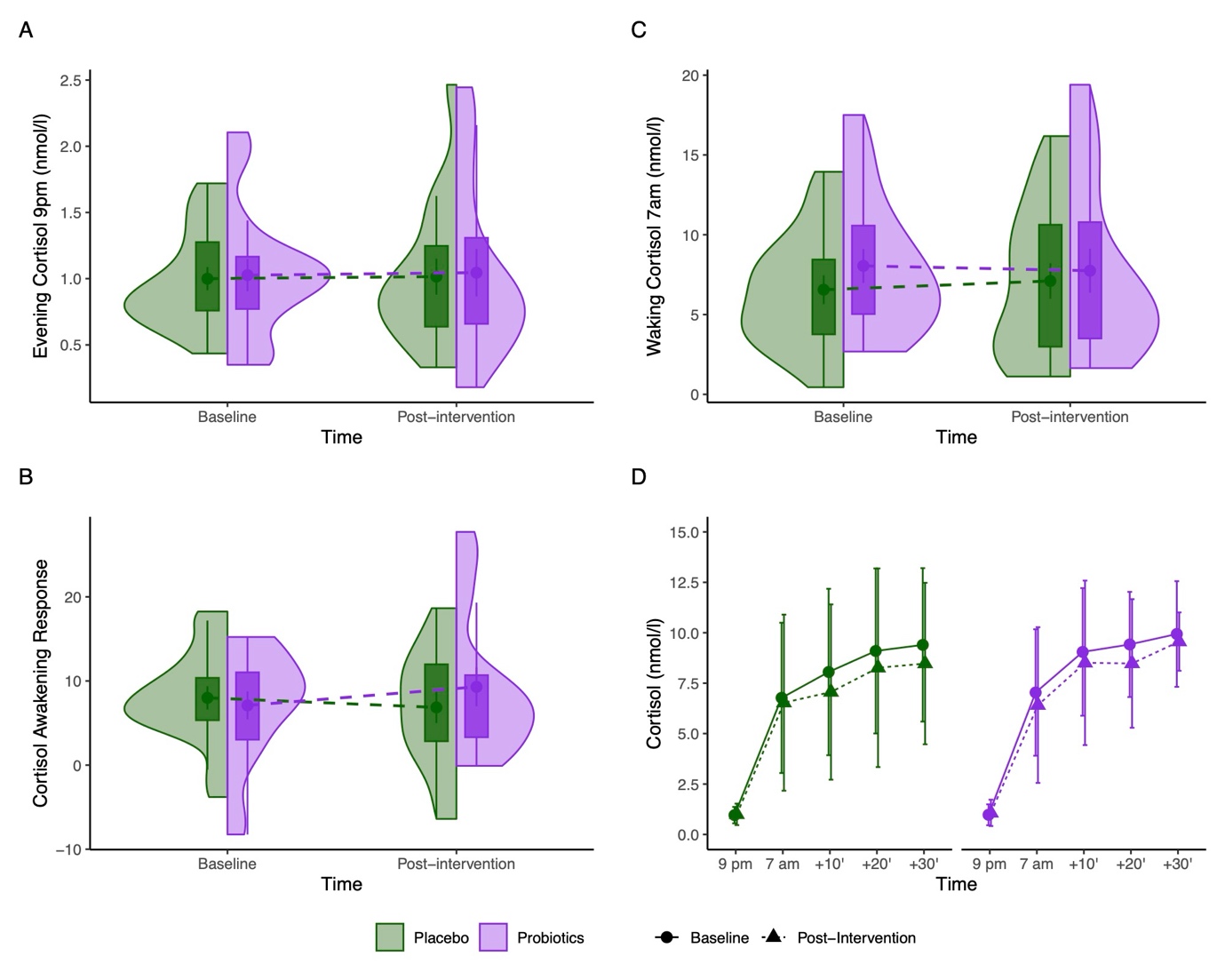
**

**sFigure 2.** Saliva-based cortisol measures in the probiotics and placebo groups from baseline to post-intervention (week 4). Cortisol concentration in A and C are presented as boxplots of Median [IQR], with Mean (SE). The cortisol awakening response (AUC_i_) in B is presented as boxplots of Median [IQR], with Mean (SE). Cortisol concentrations in D are presented as Mean (SE).

**
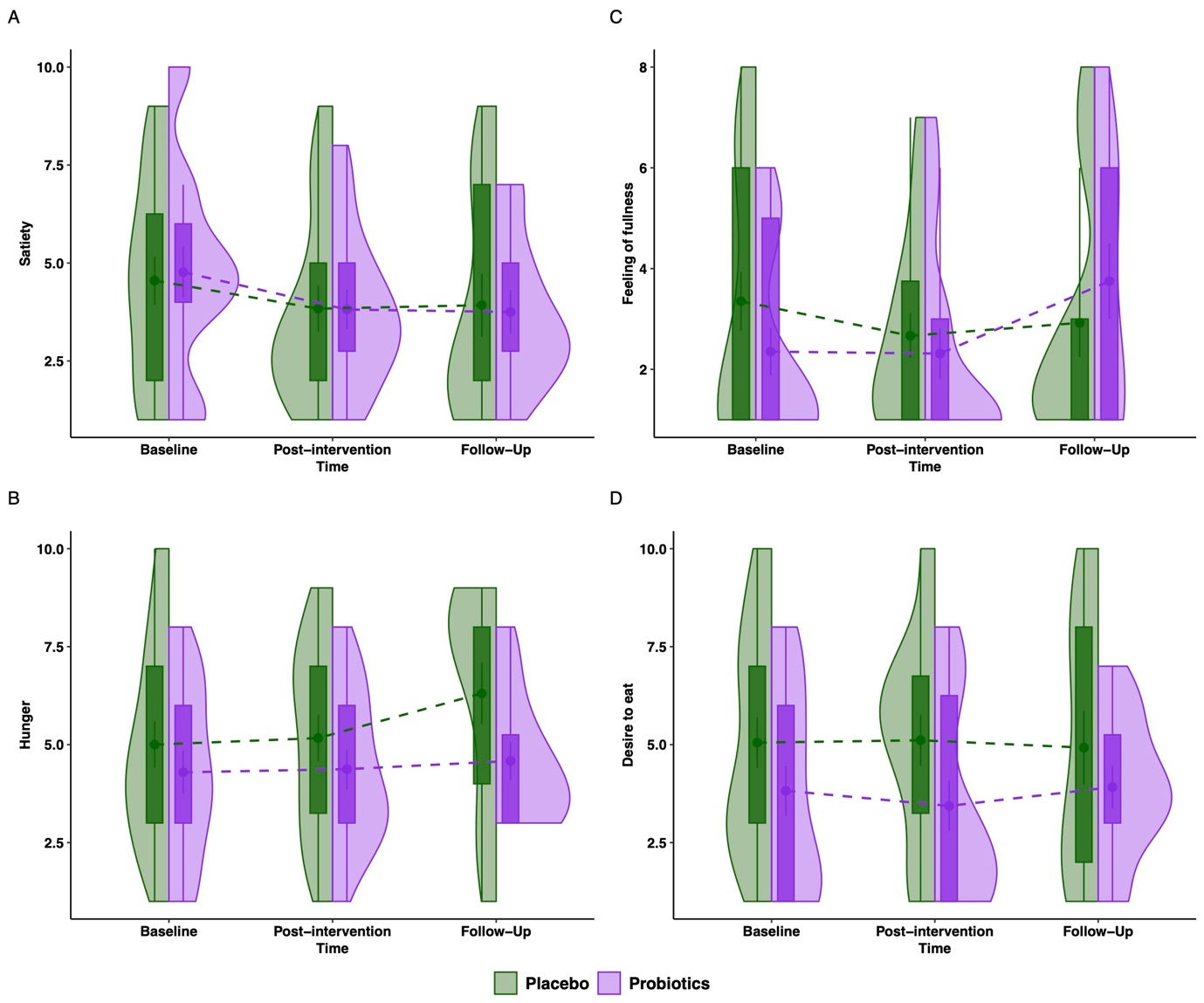
**

**sFigure 3.** Subjective appetite ratings in the probiotics and placebo groups from baseline to post-intervention (week 4) and follow-up assessment (week 8). Appetite scores are presented as boxplots of Median [IQR], with Mean (SE) and anchored by the descriptions “not at all” = 0, “extremely” = 10.

**Supplementary References**

1 World Health Organization. WHO Collaborating Centre for Drug Statistics Methodology: ATC classification index with DDDs, 2020. 2019.

2 Schaub AC, Schneider E, Vazquez-Castellanos JF, Schweinfurth N, Kettelhack C, Doll JPK *et al.* Clinical, gut microbial and neural effects of a probiotic add-on therapy in depressed patients: a randomized controlled trial. *Translational Psychiatry 2022 12:1* 2022; **12**: 1–10.

3 Yamanbaeva G, Schaub AC, Schneider E, Schweinfurth N, Kettelhack C, Doll JPK *et al.* Effects of a probiotic add-on treatment on fronto-limbic brain structure, function, and perfusion in depression: Secondary neuroimaging findings of a randomized controlled trial. *J Affect Disord* 2023; **324**: 529–538.

4 Schneider E, Doll JPK, Schweinfurth N, Kettelhack C, Schaub AC, Yamanbaeva G *et al.* Effect of short-term, high-dose probiotic supplementation on cognition, related brain functions and BDNF in patients with depression: a secondary analysis of a randomized controlled trial. *J Psychiatry Neurosci* 2023; **48**: E23–E33.

5 Martin M. Cutadapt removes adapter sequences from high-throughput sequencing reads. *EMBnet J* 2011; **17**: 10–12.

6 Wingett SW, Andrews S. FastQ Screen: A tool for multi-genome mapping and quality control. *F1000Res* 2018; **7**: 1338.

7 Davis MPA, van Dongen S, Abreu-Goodger C, Bartonicek N, Enright AJ. Kraken: a set of tools for quality control and analysis of high-throughput sequence data. *Methods* 2013; **63**: 41–49.

8 Dobin A, Davis CA, Schlesinger F, Drenkow J, Zaleski C, Jha S *et al.* STAR: ultrafast universal RNA-seq aligner. *Bioinformatics* 2013; **29**: 15–21.

9 Anders S, Pyl PT, Huber W. HTSeq—a Python framework to work with high-throughput sequencing data. *Bioinformatics* 2015; **31**: 166–169.

10 Love MI, Huber W, Anders S. Moderated estimation of fold change and dispersion for RNA-seq data with DESeq2. *Genome Biol* 2014; **15**: 1–21.

11 Langfelder P, Horvath S. WGCNA: An R package for weighted correlation network analysis. *BMC Bioinformatics* 2008; **9**: 1–13.

12 Wu T, Hu E, Xu S, Chen M, Guo P, Dai Z *et al.* clusterProfiler 4.0: A universal enrichment tool for interpreting omics data. *Innovation (Cambridge (Mass))* 2021; **2**. doi:10.1016/J.XINN.2021.100141.
